# Endobronchial valves placement for persistent air leaks in critically ill patients: chest tube removal and competing risk of death

**DOI:** 10.3389/fmed.2026.1905196

**Published:** 2026-07-28

**Authors:** Antonio Moretti, Luca Tabbì, Roberto Tonelli, Francesco Livrieri, Matteo Delle Vergini, Bianca Beghé, Enrico M. Clini, Alessandro Marchioni

**Affiliations:** 1 Respiratory Diseases Unit, Department of Medical and Surgical Sciences, University Hospital of Modena, University of Modena Reggio Emilia, Modena, Italy; 2 Residency School of Respiratory Diseases, University of Modena and Reggio Emilia, Modena, Italy

**Keywords:** alveolar-pleural fistula, chest tube drainage, critically ill patients, endobronchial valves, persistent air leaks

## Abstract

**Background:**

Persistent air leaks due to alveolar–pleural or peripheral bronchiolar–pleural fistulas remain challenging in critically ill patients requiring respiratory support. We assessed the effectiveness and safety of endobronchial valve placement in this setting, accounting for the competing risk of death.

**Methods:**

We conducted a single-center, retrospective cohort study of consecutive critically ill adults requiring invasive mechanical ventilation or high-flow nasal oxygen for acute respiratory failure who underwent endobronchial valve placement for alveolar–pleural or peripheral bronchiolar–pleural fistulas. The primary outcome was time to chest tube removal, analyzed using a competing risk analysis in which in-hospital death was treated as the competing event. Secondary outcomes included in-hospital mortality, procedural safety, and exploratory analyses of the response to early post-procedural air leaks.

**Results:**

A total of 38 patients were included. The cumulative incidence of chest tube removal reached 60.5, 71.1, and 94.7% by days 10, 14, and 20, respectively, after endobronchial valve placement; the corresponding cumulative incidence of in-hospital death was 2.6% by day 14. The median time to chest tube removal was 7.5 days. In Fine–Gray models, adjusted for clinically relevant covariates, older age was found to be associated with a lower incidence of chest tube removal (subdistribution hazard ratio [sHR], 0.96 per year; 95% CI, 0.93–0.98; *p* = 0.002), whereas pneumomediastinum was associated with a higher incidence of chest tube removal (sHR, 2.36; 95% CI, 1.12–4.97; *p* = 0.023). In-hospital mortality was 10.5%, and no procedure-related life-threatening adverse events or urgent valve removals occurred.

**Conclusion:**

In critically ill patients with persistent air leaks, endobronchial valve placement was associated with rapid chest tube removal and a low early competing risk of death. These findings support further prospective evaluation of this approach in high-risk patients.

## 1 Background

Alveolar–pleural fistulas (APFs) and peripheral bronchiolar–pleural fistulas (PBPs) are abnormal communications between the pulmonary parenchyma distal to a segmental bronchus and the pleural space (1). They are commonly associated with pneumothorax and persistent air leaks (PALs), both of which represent challenging clinical scenarios. The etiology of APFs/PBPs is heterogeneous and includes spontaneous pneumothorax, particularly in patients with underlying parenchymal lung disease (e.g., emphysema or chronic obstructive pulmonary disease), lung resection during thoracic surgery, chest trauma, necrotizing pneumonia, and iatrogenic injuries related to invasive diagnostic procedures (e.g., transbronchial or percutaneous lung biopsies) (2).

In critically ill patients, mechanical ventilation (MV) and barotrauma may precipitate or perpetuate APF, thereby complicating air leak control and compromising the ability to maintain adequate alveolar ventilation (3). Approximately 10–30% of patients with Acute Respiratory Distress Syndrome develop APF or bronchopleural fistula, and up to 2% experience PALs (4, 5). Moreover, MV, by increasing transpulmonary pressure and respiratory rate, may impair spontaneous fistula closure, thereby making these conditions difficult to manage.

A refractory APF or PBP with PAL typically requires tube thoracostomy and is generally treated surgically or with endobronchial interventions, such as endobronchial valve (EBV) placement, in patients who are not eligible for surgery. Despite the recent European Respiratory Society/European Association for the Cardio-Thoraicc Society/European Society of Thoracic Surgery clinical practice guidelines not providing specific recommendations for or against EBV placement in these patients who are unfit for surgery, several small-sample studies have reported encouraging results with this technique (6). Nevertheless, evidence in critically ill patients remains scarce, and concerns persist regarding both the effectiveness and the potential risk of respiratory deterioration associated with EBV placement.

This study aimed to evaluate the efficacy and safety of EBV placement in patients with respiratory failure who required invasive and non-invasive advanced respiratory support, addressing an important knowledge gap in this high-risk population.

## 2 Materials and methods

### 2.1 Study design and setting

We conducted a single-center, retrospective cohort study of consecutive patients treated with EBVs (Zephyr^®^ EBV^®^, Pulmonx Corporation, Redwood City, CA, USA) for APFs/PBPs at the Diagnostic and Interventional Bronchoscopy Unit, University Hospital of Modena, between January 2020 and June 2025. Clinical, endoscopic, and radiological variables were prospectively collected in a dedicated registry, ensuring systematic and predefined data acquisition despite the retrospective design of the analysis.

### 2.2 Patient selection

Consecutive critically ill adults (≥18 years) requiring advanced respiratory support (including invasive and non-invasive mechanical ventilation or high-flow nasal oxygen) for acute respiratory failure and undergoing EBV placement for APF/PBP were all included. Given the heterogeneity of respiratory support modalities, the type of support at the time of EBV placement was recorded and considered in the analysis. APF/PBP was defined as a persistent air leak originating from the lung parenchyma distal to a segmental bronchus, supported by imaging and bronchoscopic confirmation, and was managed with pleural drainage. EBV placement was performed at the discretion of the treating team based on leak volume, respiratory and hemodynamic status, and leak duration. Patients were excluded if they had a concomitant proximal bronchopleural fistula (e.g., bronchial stump dehiscence or the visible segmental/lobar bronchial defect) or if they did not require respiratory support at the time of EBV placement. Patients with hemodynamic instability and an expected survival of <24 h were also excluded from the study.

### 2.3 Pre-procedural management

Before EBV placement, patients received pleural drainage and optimization of respiratory support as standard-of-care management for PALs. Chest tube management followed institutional protocols and was recorded for each patient. Generally, we advised treating physicians to avoid applying or to stop wall suction after a PAL was established, as the literature reports more favorable outcomes (1), favoring a water-seal system. We used a digital monitoring system for air leaks when available, including during the bronchial occlusion test. Drainage clamping was typically performed only after the leak was arrested or significantly reduced, allowing for radiographic confirmation after 12–24 h.

EBV placement was considered after the failure of conservative management for air leaks lasting longer than 5 days or performed on an urgent basis in cases of high-volume leaks with a severe impact on respiratory exchanges or on the effectiveness of mechanical ventilation.

### 2.4 EBV procedure and localization strategy

EBV placement was performed by experienced interventional bronchoscopists under general anesthesia with a flexible bronchoscopy via an endotracheal tube or laryngeal mask. The target segment(s) were identified using a standardized localization strategy. For all patients, a bronchial occlusion test was performed using a balloon catheter, with a “positive” test defined as the immediate cessation or a significant reduction in the air leak from the drainage system, as assessed by direct visual observation of bubbling reduction in the water seal chamber during occlusion or by the digital monitoring system. Unfortunately, this assessment was available for only a few patients included in this study; therefore, we cannot draw any conclusions on its role in this setting. In cases with inconclusive/negative occlusion tests, or with high suspicion of multifocal leaks, EBVs were placed in the most likely involved segment(s) according to chest CT findings and, when applicable, according to the location where airflow reduction was greatest during the occlusion test; this information was explicitly recorded. The number of treated segments/lobes, the number of valves, and the need for multilobar treatment (≥2 lobes) were documented. Post-procedural management included a chest radiograph performed within 2 h after the procedure, continued pleural drainage management according to protocol, and adjustment of ventilatory settings as clinically indicated.

### 2.5 Data collection

Demographics, comorbidities (assessed using the Charlson Comorbidity Index), etiology of APF/PBP, baseline respiratory support (MV/High-flow Nasal Oxygen), gas exchange indices, radiological findings (including pneumomediastinum), procedural details (treated lobes and number of valves), and complications were extracted from the registry. Follow-up included the timing of chest tube removal, the duration of the air leak, in-hospital outcomes, and the mortality rate.

### 2.6 Outcomes

The *primary outcome* was the time from EBV placement to chest tube removal, with in-hospital death considered a competing event.

The *secondary outcomes* included in-hospital mortality, safety, including post-procedural adverse events, and exploratory analyses according to post-procedural air leak response. Air leak response was classified according to the first post-procedural assessment using Cerfolio’s criteria*: complete resolution* was defined as immediate cessation of the air leak; *partial resolution* as a ≥ 2-point reduction in Cerfolio grade; otherwise, it was defined as no improvement. Complete or partial resolution was grouped as “air leak improvement.”

### 2.7 Statistical analysis

Continuous variables are reported as median (interquartile range: IQR) and categorical variables as *n* (%). Baseline characteristics were described overall and by prespecified groups, which were defined by chest tube removal time through a median-based stratification (≤13 vs. >13 days), using standardized mean differences (SMDs) as the primary measure of between-group imbalance; hypothesis tests were provided as exploratory. Competing risk methods were used for the primary outcome. Cumulative incidence functions (CIFs) were estimated for chest tube removal (the event of interest) and in-hospital death (the competing event), with point estimates and 95% CIs at prespecified time points (e.g., days 10, 14, and 20). Associations with chest tube removal were assessed using Fine–Gray subdistribution hazard models (sHRs), adjusting for a limited set of clinically relevant covariates selected *a priori* (age, mechanical ventilation, PaO₂/FiO₂ (P/F) ratio ≤200, pulmonary fibrosis, pneumomediastinum, and multilobar treatment [≥2 lobes]). The results are presented as sHRs with 95% CIs. Missing data were handled using an available-case analysis without imputation. Given the limited sample size, these analyses should be considered exploratory. Analyses were performed in R using the competing risk and survival packages.

## 3 Results

### 3.1 Study population

A total of 38 consecutive critically ill patients undergoing EBV placement for PAL were included. Baseline characteristics are summarized in Table 1. The groups showed broadly comparable baseline profiles, with standardized mean differences generally ranging from small to moderate. Overall, 12 patients (32%) had a negative or inconclusive bronchial occlusion test, while only one patient was treated on an urgent basis after having an air leak for 4 days before EBV placement.

**Table 1:** Baseline characteristics of the overall cohort (*N* = 38).

Characteristic^1^	*N* ^1^	Overall (*N* = 38)^1^
Age (years), Median [Q1, Q3]	38	68 [59, 74]
Age ≥68 years, *n* (%)	38	21 (55%)
P/*F* ≤ 200, *n* (%)	38	26 (68%)
Mechanical ventilation, *n* (%)	38	12 (32%)
Charlson index ≥5, *n* (%)	38	29 (76%)
COPD, *n* (%)	38	17 (45%)
Emphysema, *n* (%)	38	20 (53%)
Fibrosis, *n* (%)	38	5 (13%)
COVID-19, *n* (%)	38	9 (24%)
FEV1 < 70% predicted, *n* (%)	16	5 (31%)
Missing		22
Air leak duration pre-EBV (days), Median [Q1, Q3]	38	20 [11, 35]
Pneumomediastinum, *n* (%)	38	11 (29%)
Cerfolio classification, *n* (%)	38	
3		14 (37%)
4		24 (63%)
Iatrogenic cause, *n* (%)	38	24 (63%)
Infective cause, *n* (%)	38	7 (18%)
Other lung diseases, *n* (%)	38	
No		30 (79%)
Yes		8 (21%)
Number of valves, *n* (%)	35	
1		17 (49%)
2		11 (31%)
3		4 (11%)
4		1 (3%)
5		1 (3%)
6		1 (3%)
Missing		3
Lobes treated, *n* (%)	38	
1		28 (74%)
2		9 (24%)
3		1 (3%)
Treated lobe (region), *n* (%)	32	
Upper lobe		14 (44%)
Middle/lingula		8 (25%)
Lower lobe		10 (31%)
Missing		6
Valve size (group), *n* (%)	38	
4.0		16 (42%)
4.5		1 (3%)
5.0		7 (18%)
5.5		12 (32%)
Mixed		2 (5%)
Bronchial blocker, *n* (%)	38	26 (68%)

^1^Values are median [IQR] or *n* (%). Percentages computed among non-missing values.

Values are median (IQR) or *n* (%). Percentages are calculated among non-missing observations; the “N” column reports the number of non-missing values per variable. Missing data were not imputed. “Cerfolio classification” denotes the grade of persistent air leak severity (higher grades indicate more severe/complex leaks).

EBV, endobronchial valve; IQR, interquartile range; COPD, chronic obstructive pulmonary disease; P/F, PaO₂/FiO₂ ratio; FEV1, forced expiratory volume in 1 s; CCI, Charlson Comorbidity Index.

### 3.2 Primary outcome

**Figure 1:**
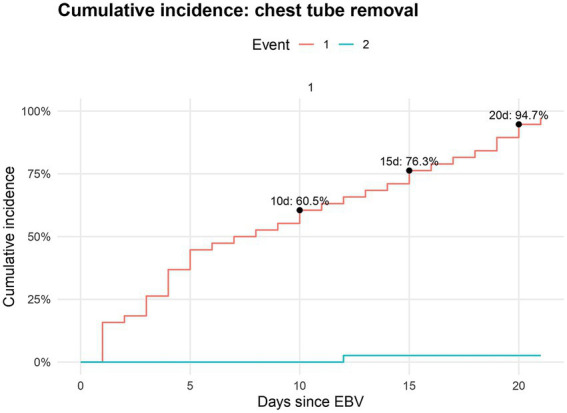
Cumulative incidence of chest tube removal following EBV placement estimated using competing risk methodology, with death treated as a competing event. EBV, endobronchial valve.

The cumulative incidence of chest tube removal reached 60.5, 71.1, and 94.7% by days 10, 14, and 20, respectively, after EBV placement. The cumulative incidence of in-hospital death by day 14 was 2.6% (95% CI: 0–7.9). The majority of chest tube removals occurred within the first 2 weeks after EBV placement (Figure 1).

**Figure 2:**
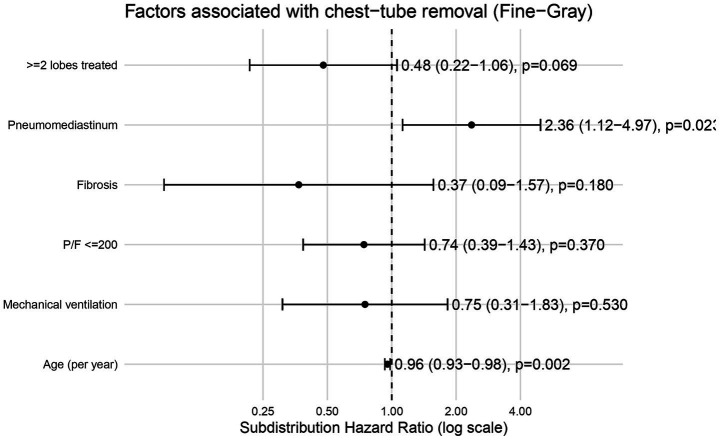
Factors associated with chest tube removal after endobronchial valve placement (Fine–Gray model). Subdistribution hazard ratios (sHRs) with 95% confidence intervals from a multivariable Fine–Gray model (event of interest: Chest tube removal; competing event: In-hospital death). Covariates included age (per year), mechanical ventilation at EBV placement, P/F ratio ≤200, pulmonary fibrosis, pneumomediastinum, and ≥2 treated lobes. The vertical line marks sHR = 1. *p*-values were derived from the Wald tests. sHR, subdistribution hazard ratio.

In the exploratory adjusted Fine–Gray analyses, increasing age was independently associated with a lower incidence of chest tube removal (sHR, 0.96 per year, 95% CI: 0.93–0.98; *p* = 0.002) (Table 2; Figure 2). In contrast, pneumomediastinum at baseline was associated with a higher incidence of chest tube removal (sHR, 2.36, 95% CI: 1.12–4.97; *p* = 0.023). Multilobar treatment (≥2 lobes) showed a borderline association with a lower incidence of chest tube removal (sHR, 0.48, 95% CI: 0.22–1.06; *p* = 0.069). Mechanical ventilation, PaO₂/FiO₂ ratio ≤200, and pulmonary fibrosis were not significantly associated with the outcome. Even stratified cumulative incidence curves suggested a higher probability of chest tube removal among patients with pneumomediastinum, although this was not significant in unadjusted analyses (Gray’s test *p* = 0.42) (Supplementary Figure S1).

**Table 2:** Factors associated with chest tube removal after EBV (fine–gray model).

Term	sHR (95% CI)	*p*-value
Age (per year)	0.96 (0.93–0.98)	0.002
Mechanical ventilation	0.75 (0.31–1.83)	0.530
P/F ≤ 200	0.74 (0.39–1.43)	0.370
Fibrosis	0.37 (0.09–1.57)	0.1 80
Pneumomediastinum	2.36 (1.12–4.97)	0.023
≥2 lobes treated	0.48 (0.22–1.06)	0.069

Results from a Fine–Gray subdistribution hazards model with in-hospital death as the competing event. The outcome was time to first chest tube removal, measured in days from the EBV procedure (Day 0). Estimates are subdistribution hazard ratios (sHRs) with 95% confidence intervals (CIs) and two-sided *p*-values; sHR > 1 indicates a higher cumulative incidence of chest tube removal over time. Covariates included *a priori*: age (per year), mechanical ventilation at EBV, PaO_2_/FiO_2_ ratio ≤200, pulmonary fibrosis, pneumomediastinum, and ≥2 lobes treated. Analyses used complete-case data; percentages were calculated among non-missing values.

EBV, endobronchial valve; sHR, subdistribution hazard ratio; CI, confidence interval.

Model: Fine–Gray (failcode = 1: drain removal; competing event = in-hospital death). sHR, subdistribution hazard ratio.

### 3.3 Secondary outcomes

**Figure 3:**
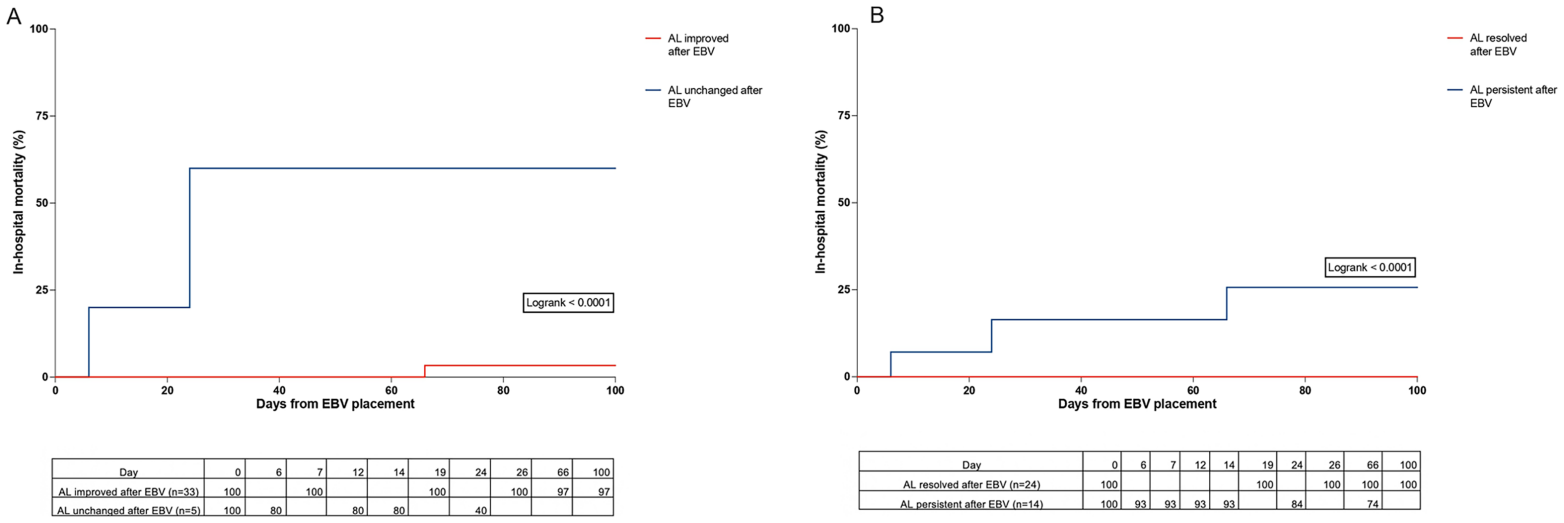
In-hospital mortality since EBV placement by early air leak response (panel **A**: leak improved; panel **B**: leak resolved) (Kaplan–Meier; in-hospital death as the event; discharge censored).

**Figure 4:**
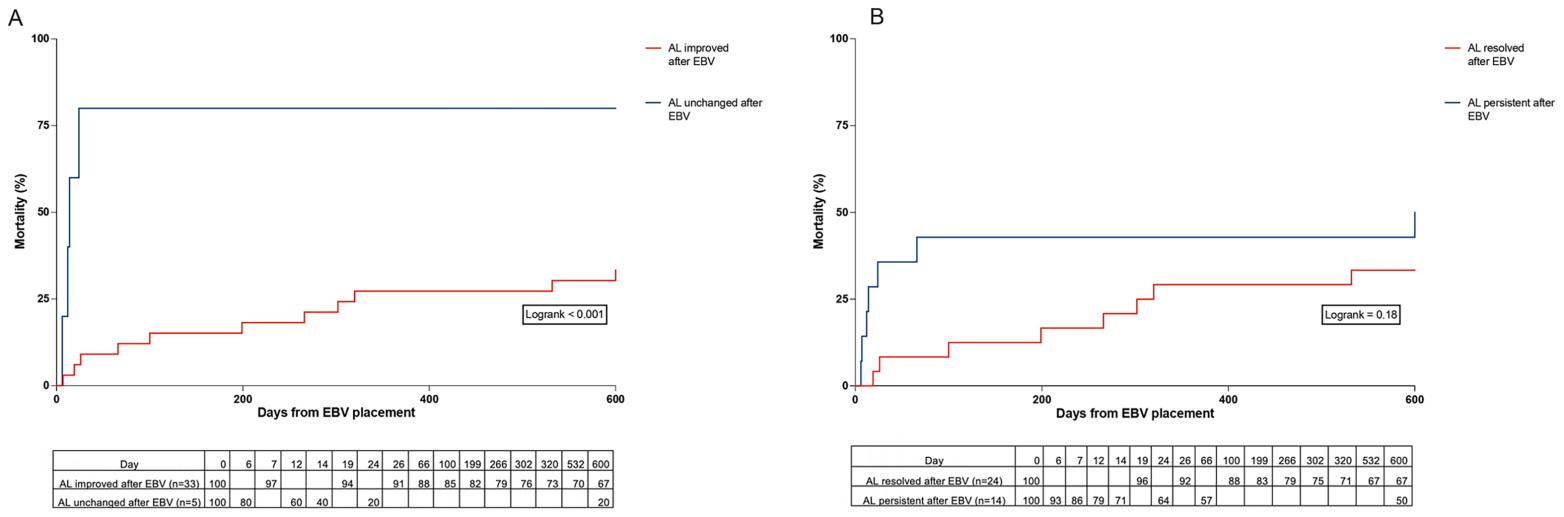
Overall survival since EBV placement by early air leak response (Kaplan–Meier) (panel **A**: leak improved; panel **B**: leak resolved).

In-hospital mortality was 10.5% (4/38), with few deaths occurring early after the procedure. Kaplan–Meier estimates of time to chest tube removal were consistent with competing risk analyses (median 7.5 days; Supplementary Figure S2). In the exploratory analyses, in-hospital mortality was lower among patients with air leak improvement than among those without improvement (log-rank *p* < 0.0001) (Figure 3A). Similar findings were observed for complete resolution vs. air leak persistence (Figure 3B). During the follow-up, overall mortality was 36.8% (14/38). Survival remained higher among patients with air leak improvement (log-rank *p* < 0.001) (Figure 4A), whereas no difference was observed between complete resolution and persistence (log-rank *p* = 0.18) (Figure 4B).

No procedure-related life-threatening adverse events or cases of acute respiratory deterioration requiring urgent valve removal were observed. Detailed safety and clinical outcomes are reported in Table 3.

**Table 3:** Post-procedural adverse events after EBV.

Adverse events	*n* (%)
Post-obstructive pneumonia	8/38 (21.1%)
Valve dislocation	0/38 (0.0%)
Hypersecretion	15/38 (39.5%)
Hemoptysis	2/38 (5.3%)
Granulation tissue	12/38 (31.6%)
Pneumothorax recurrence after valve removal	0/6 (0.0%)
In-hospital mortality	4/38 (10.5%)
All-cause mortality	14/38 (36.8%)

Summary of safety through follow-up. Binary outcomes, including the adverse events listed in the table, are presented as *n*/*n* (%), where *N* is the number of cases with available data. Day 0 is the date of the EBV procedure.

Among patients with available follow-up data (n = 28), the median follow-up duration after EBV placement was 206 days (IQR: 29.3–308). Valve removal was performed in 15 patients after a median of 7 months (IQR: 3.5–11.5) following implantation.

## 4 Discussion

In critically ill patients, EBV placement was associated with rapid chest tube removal and a low early competing risk of death, supporting the feasibility of this approach in this high-risk population with complications related to bronchoscopic intervention.

### 4.1 Key findings

In critically ill patients, EBV placement for PAL was associated with a high rate of chest tube removal within a relatively short timeframe (within the first 2 weeks) and a low early incidence of the competing event of in-hospital death. These findings suggest the presence of a therapeutic window in which bronchoscopic intervention may promote air leak resolution without substantially increasing the risk of early mortality.

In addition, the association between pneumomediastinum and a higher incidence of chest tube removal warrants further speculative discussion. While pneumomediastinum is typically associated with severe disease, our data may identify a subgroup of patients in whom PAL is predominantly driven by dynamic pressure-related mechanisms. In this context, reduction in regional airflow and pressure gradients with EBV placement could provide a potential pathophysiological explanation for the observed association. This hypothesis warrants confirmation in future studies.

Finally, increasing age was associated with a lower probability of chest tube removal over time, likely reflecting reduced physiological reserve and impaired tissue repair capacity. Notably, early improvement in air leaks after EBV placement was associated with more favorable clinical trajectories.

### 4.2 Rationale for EBV placement in patients with persistent air leaks

EBV is the most widely adopted bronchoscopic approach for the management of PAL (7).

Its physiological effect is multifactorial, combining a reduction in regional airflow, a decrease in the trans-fistula pressure gradient, and the induction of controlled lobar atelectasis. Together, these mechanisms reduce alveolar distension and mechanical stress at the level of the fistula, thereby promoting a state of “regional lung rest” that facilitates spontaneous healing processes, including fibrin deposition, fibroblast activation, and epithelial repair.

In addition to this mechanical role, EBVs can be conceptualized as modulators of local biomechanical forces that are critical for maintaining fistula patency. This aspect is particularly relevant in mechanically ventilated patients, in whom positive pressure ventilation can perpetuate the air leak through sustained high airway pressures and repeated mechanical stress on the injured lung parenchyma. By functionally isolating the affected region, EBVs can interrupt this cycle and create a more favorable environment for tissue repair.

In this context, the presence of pneumomediastinum may provide additional pathophysiological insights. Pneumomediastinum is typically interpreted as a marker of alveolar rupture due to elevated intra-alveolar pressure and pressure gradients between the lung parenchyma and surrounding structures. However, it may also identify a subset of patients in whom the air leak is primarily driven by dynamic pressure-related mechanisms rather than fixed structural disruption. In these cases, the underlying pathophysiology may be particularly amenable to modulation through bronchial occlusion strategies. Consistent with this hypothesis, the association observed in our study between pneumomediastinum and a higher incidence of chest tube removal suggests that EBV-induced reduction in regional airflow and pressure gradients may be particularly effective in this setting. Supplementary Figure S3 illustrates two representative CT presentations, one with pneumothorax and the other with pneumomediastinum, highlighting the heterogeneity of disease patterns and the potential relevance of pressure-related mechanisms.

Emerging experimental data also suggest that hypercapnia may impair lung healing by inhibiting key pathways involved in cell migration, such as CXCL12 signaling and Rac1-GTPase activity (8). Therefore, impaired ventilation and CO2 retention may further contribute to delayed fistula closure when PAL occurs. Within this framework, EBV placement may exert indirect biological effects by improving ventilatory efficiency and mitigating factors that hinder tissue repair.

Although the efficacy of EBVs has been reported in multiple retrospective studies and summarized in recent meta-analyses, the available evidence is largely derived from stable patients without the need for advanced respiratory support (7, 9, 10). This study extends existing knowledge by specifically evaluating the role of EBVs in a high-risk population requiring mechanical ventilation or high-flow oxygen support.

### 4.3 PAL in mechanically ventilated patients and the role of EBV

In mechanically ventilated patients, PAL represents a particularly challenging clinical condition, associated with substantial morbidity and mortality (11). The interaction between positive-pressure ventilation and fistula dynamics creates a self-perpetuating cycle in which elevated airway pressures increase airflow across the fistula, thereby impairing spontaneous closure and compromising effective ventilation (12).

From a physiological perspective, flow through an alveolar–pleural or bronchopleural fistula is driven by the pressure gradient between the airway and the pleural space and is influenced by factors such as airway pressure, pleural pressure, and fistula geometry. Thus, higher levels of positive end-expiratory pressure (PEEP), while often necessary to maintain alveolar recruitment, may paradoxically exacerbate the air leak by increasing the trans-fistula pressure gradient and promoting continuous airflow diversion. This phenomenon contributes to ineffective ventilation, prolonged respiratory support, and poor clinical outcomes (13).

Exploratory analyses did not demonstrate a robust association between pre-procedural air leak duration and time to chest tube removal. Spearman’s correlation was negligible (rho = −0.03, *p* = 0.89), whereas the apparent moderate Pearson correlation was largely driven by extreme observations and disappeared after the exclusion of outliers (r = 0.15, *p* = 0.45). Similarly, no relevant difference in median time to chest tube removal was observed between patients undergoing earlier and later EBV placement based on the cohort median pre-procedural air leak duration. These exploratory findings may suggest that, once effective bronchial occlusion is achieved, the duration of the previous persistent air leak becomes less relevant to post-procedural recovery. However, these analyses should be interpreted cautiously given the limited sample size and the presence of highly skewed observations.

Within this framework, EBV placement offers a mechanistically coherent approach by directly targeting the airflow driving the fistula. By reducing regional ventilation and limiting the pressure gradient across the injured lung segment, EBVs may mitigate the mechanical forces that sustain the air leak and facilitate tissue repair.

Nevertheless, the application of EBVs in critically ill patients remains debated. Theoretical concerns include the potential worsening of gas exchange due to increased ventilation–perfusion mismatch and the unpredictable effects on lung compliance, particularly in patients with severely compromised respiratory mechanics. Notably, these concerns are largely based on physiological considerations rather than robust clinical evidence.

To our knowledge, this study is among the first to suggest the early benefits and safety of EBV placement in critically ill patients with PAL. The inclusion of patients receiving different forms of advanced respiratory support reflects the real-world heterogeneity of critically ill patients with persistent air leaks. While this may introduce variability, it enhances the clinical applicability of the findings across a broad spectrum of disease severity. Previous reports on EBV use in critically ill patients have suggested a potential role in facilitating weaning from mechanical ventilation and clinical stabilization (14–16) based on small case series. Our study builds on this evidence and supports the role of EBVs as a feasible therapeutic option in this challenging clinical scenario.

However, the overall results should be interpreted cautiously, as post-treatment stratification does not allow causal inference and may primarily reflect underlying differences in disease severity and reversibility. Indeed, it is possible that the early response to EBV identifies a subgroup of patients with more favorable underlying physiopathology.

### 4.4 Limitations

The limitations of this study should also be acknowledged. First, the retrospective design limits the strength of the conclusions and allows only for an exploratory interpretation of the findings. Second, this is a single-center study conducted in a high-volume referral center with substantial expertise in EBV placement, which may limit the generalizability of the results to less experienced centers. Third, due to the absence of a control group, our study cannot determine whether EBV placement was superior to conservative management or surgery. However, according to clinical experience and previous reports, the time to tube removal in successful cases appears shorter than that observed with PAL resolution with a chest drain alone (1, 2). In addition, analyses based on post-treatment variables, such as early air leak improvement, are inherently subject to post-treatment bias and should be interpreted as descriptive rather than predictive. Given the relatively small sample size, the Fine–Gray model includes several covariates despite the limited number of patients and events, and multivariable analyses may be prone to overfitting and should be interpreted as exploratory.

Finally, the lack of detailed physiological data, including respiratory system compliance and gas exchange parameters before and after EBV placement, limits our ability to directly assess the mechanical and functional impact of the intervention.

## 5 Conclusion

EBV placement is a feasible and safe option for the management of PAL, even in critically ill patients. This endoscopic strategy seems to allow relatively rapid chest tube removal. However, the hypothesis-generating findings of this study should be interpreted cautiously and require future prospective controlled studies to confirm the results and to further define patient selection criteria.

## Data Availability

The raw data supporting the conclusions of this article will be made available by the authors, without undue reservation.

## Supplementary material

Supplementary Figure S1 Cumulative incidence of chest-tube removal by pneumomediastinum status. Cumulative incidence functions (Fine–Gray) for chest-tube removal, stratified by presence of pneumomediastinum at baseline; in-hospital death treated as a competing event. No global difference between curves was observed (Gray’s test *p* = 0.423). CIF, cumulative incidence function; EBV, endobronchial valves; CI, confidence interval.

Supplementary Figure S2 Time to chest tube removal after endobronchial valve (EBV) placement (Kaplan–Meier). Kaplan–Meier curve showing the proportion of patients with the chest tube still in place over time (shaded band = 95% CI), with in-hospital death treated as censoring. The median time to removal was 7.5 days. The risk table reports the number at risk at selected time points. (The complementary quantity, 1 − KM(t), corresponds to the cumulative probability of chest-tube removal by time t.).

Supplementary Figure S3 Two representative presentations OF pal at CT scan. **(A)** illustrates a patient affected with pneumothorax; **(B)** illustrates a patient with pneumomediastinum.
