# Supplementary material for: Endobronchial valves placement for persistent air leaks in critically ill patients: chest tube removal and competing risk of death
Source: Front Med (Lausanne). 2026 Jul 28;13:1905196. doi: 10.3389/fmed.2026.1905196 (PMC13457204; doi:10.3389/fmed.2026.1905196)

Cumulative incidence of chest−tube removal by pneumomediast

Gray's test p = 0.423

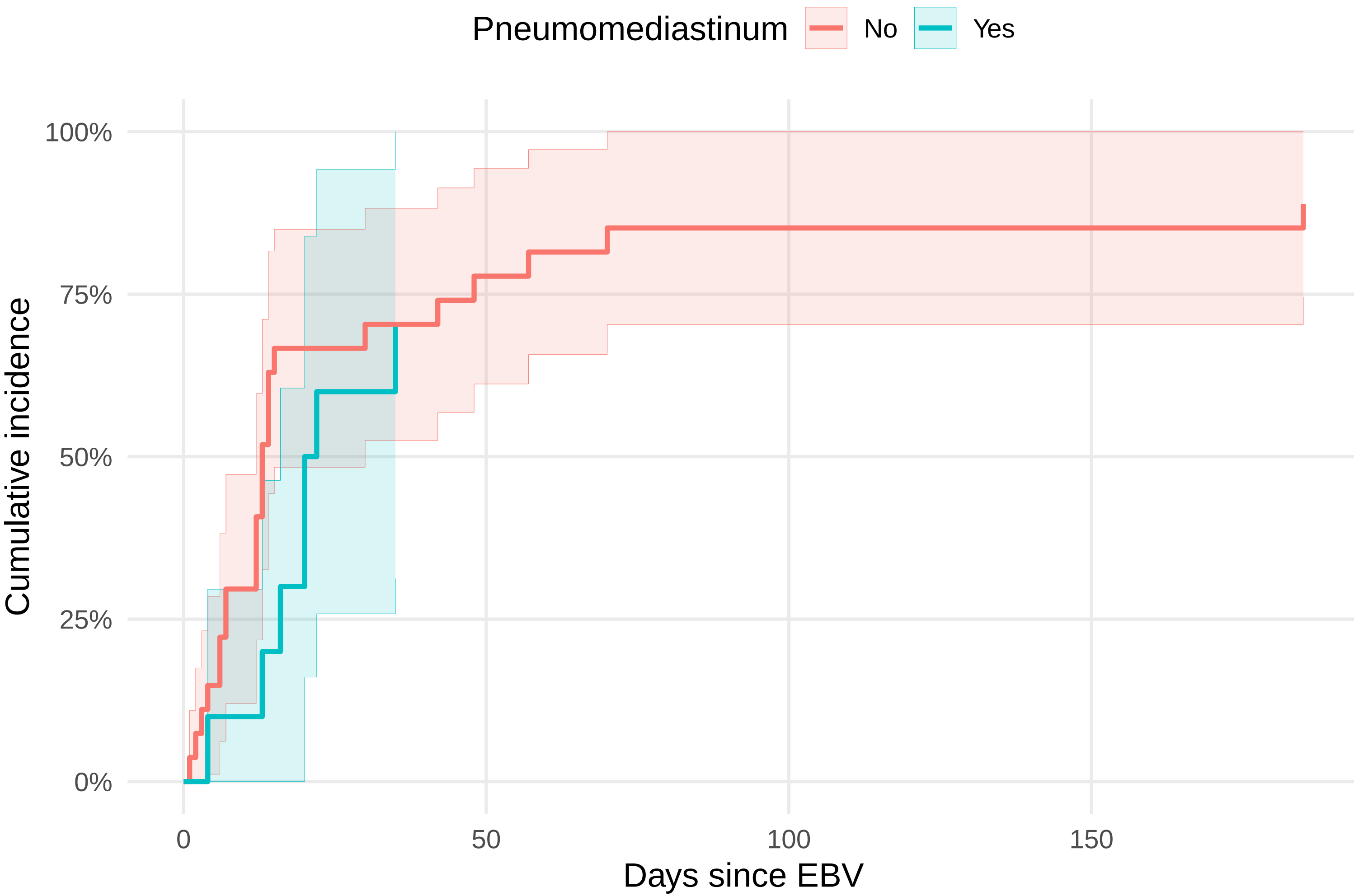

Supplement: Supplementary file 1 [file Data_Sheet_1.PDF]
