# Supplementary material for: Endobronchial valves placement for persistent air leaks in critically ill patients: chest tube removal and competing risk of death
Source: Front Med (Lausanne). 2026 Jul 28;13:1905196. doi: 10.3389/fmed.2026.1905196 (PMC13457204; doi:10.3389/fmed.2026.1905196)
Supplement: Supplementary file 2 [file Data_Sheet_2.PDF]

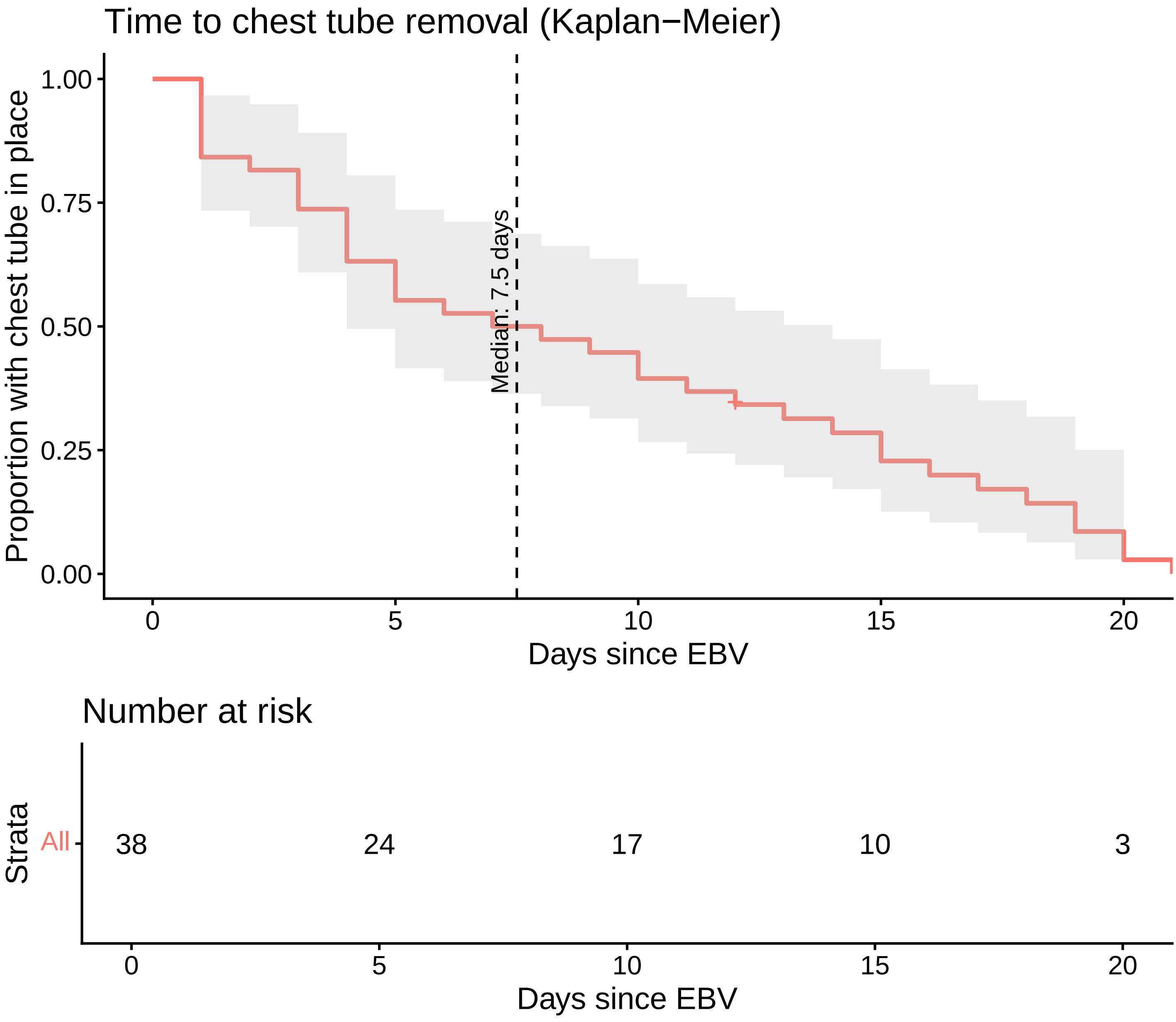
Time to chest tube removal (Kaplan−Meier)
Proportion with chest tube in place
1.00
0.75
0.50
0.25
0.00
Median: 7.5 days
0
5
10
15
20
Days since EBV
Number at risk
Strata
All
38
24
17
10
3
0
5
10
15
20
Days since EBV
